# Preventing and Managing Urinary Tract Infections: Enhancing the Role of Community Pharmacists—A Mixed Methods Study

**DOI:** 10.3390/antibiotics9090583

**Published:** 2020-09-07

**Authors:** Nathan Peiffer-Smadja, Rosalie Allison, Leah F. Jones, Alison Holmes, Parvesh Patel, Donna M. Lecky, Raheelah Ahmad, Cliodna A. M. McNulty

**Affiliations:** 1 National Institute for Health Research Health Protection Research Unit in Healthcare Associated Infections and Antimicrobial Resistance, Faculty of Medicine, Imperial College London, The Hammersmith Hospital, Commonwealth Building, Du Cane Road, London W12 0NN, UK; alison.holmes@imperial.ac.uk; 2 IAME, Université de Paris, INSERM, F-75018 Paris, France; 3 Infectious and Tropical Diseases Department, Bichat-Claude Bernard Hospital, AP-HP, F-75018 Paris, France; 4 Primary Care and Interventions Unit, Public Health England, Gloucester GL1 1DQ, UK; rosie.allison@phe.gov.uk (R.A.); Leah.jones@phe.gov.uk (L.F.J.); donna.lecky@phe.gov.uk (D.M.L.); Cliodna.McNulty@phe.gov.uk (C.A.M.M.); 5 Local Pharmaceutical Committee, Newham, London E7 8BA, UK; pmpatel01@gmail.com; 6 School of Health Sciences, City University of London, Northampton Square, London EC1V 0HB, UK

**Keywords:** pharmacist, community, urinary tract infections, leaflet, self-care, general public

## Abstract

Background: Community pharmacists are involved in antimicrobial stewardship through self-care advice and delivering medications for uncomplicated infections. Objectives: This mixed methods study aimed to identify opportunities to enhance the role of community pharmacists in the management of service users with suspected or confirmed urinary tract infection (UTI). Methods: Data collection was through a service user survey (n = 51) and pharmacist surveys and semi-structured interviews before (16 interviews, 22 questionnaires) and after (15 interviews, 16 questionnaires) trialing UTI leaflets designed to be shared with service users. Data were analysed inductively using thematic analysis and descriptive tabulation of quantitative data. Results: Twenty-five percent (n = 13/51) of service users with urinary symptoms sought help from a pharmacist first and 65% (n = 33/51) were comfortable discussing their urinary symptoms with a pharmacist in a private space. Community pharmacists were confident as the first professional contact for service users with uncomplicated UTI (n = 13/16, 81%), but indicated the lack of a specific patient referral pathway (n = 16/16, 100%), the need for additional funding and staff (n = 10/16, 62%), and the importance of developing prescription options for pharmacists (5/16, 31%). All community pharmacists reported playing a daily role in controlling antimicrobial resistance by educating service users about viral and bacterial infections and promoting a healthy lifestyle. Enhancing their role will need greater integrated working with general practices and more prescribers based in community pharmacy. Conclusion: This study suggests that community pharmacists could play a greater role in the management of uncomplicated UTI. The current reconfiguration of primary care in England with primary care networks and integrated care systems could provide a real opportunity for this collaborative working with potential learning for international initiatives.

## 1. Introduction

Urinary tract infections (UTIs) are one of the most commonly seen bacterial infection in general practice. Up to 50% of women will have a UTI in their lifetime and 30% among them will have recurring episodes [1,2]. Outcomes are usually very good as most cases resolve in 3–4 days without complications with empiric antibiotics [3]. Of all the antibiotics prescribed in primary care, 15–20% are prescribed for UTIs [4]. However, as many as 60% of women with suspected UTIs have a urine culture negative for bacteria and may not need antibiotics [5]. The prevalence of inappropriate antibiotic prescribing for UTI in primary care may be over 70% [6]. However, UTIs account for around 20% of community-acquired bacteraemia in patients admitted to an English National Health Service (NHS) Trust in 2007–08 and studies suggest that up to 50% of *Escherichia coli* bloodstream infections in England could be owing to urinary tract infections [7,8]. Furthermore, increases in the incidence of *Escherichia coli* bloodstream infections in England were mostly driven by community cases [8].

In a recent study, 95% of women consulted a health professional for their most recent UTI: 74% reported being prescribed an antibiotic, yet only 63% of these reported taking them, highlighting the need for better advice about antibiotics in the community [9]. Community pharmacists are involved daily in antimicrobial stewardship (AMS) by providing patients with self-care advice, delivering medications, and recommending over-the-counter (OTC) treatments for common infections. The U.K. 2019 5-year antimicrobial resistance (AMR) action plan states that primary care pharmacists have a critical role in reviewing prescriptions for antimicrobials and challenging those that may be inappropriate [10]. National and local campaigns advise the general public to first contact their pharmacist for healthcare advice [9]. Community-based interventions by pharmacists have the potential to control the rise of bacteraemia and to improve antimicrobial use for UTIs by increasing patient knowledge and self-care skills. Measures such as enhanced self-care, preventative care, and referral to general practitioners (GPs) when appropriate could improve the health and wellbeing of the general public. This is particularly relevant as virtually all service users with suspected or confirmed UTI visit a community pharmacy either before their GP or after to collect a prescription. Patient group directions (PGDs) allow healthcare professionals to supply and administer specified medicines to pre-defined groups of patients, without a prescription [11]. While PGD for patients with uncomplicated UTIs fulfilling specific criteria allow pharmacists to provide nitrofurantoin, thus improving patient access to early treatment [12], few guidelines have been developed to guide pharmacists in the community management of suspected or confirmed UTI. The International Pharmaceutical Federation issued in 2015 a report on the contribution of pharmacists to fight antimicrobial resistance [13]. While most interventions are targeting hospital pharmacists, some countries have issued policies for community pharmacists, such as New Zealand or Canada, where community pharmacists can prescribe first-line antibiotics for uncomplicated UTIs [14,15].

The U.K. national action plan (NAP) for AMR indicates a need to strengthen the links between primary care pharmacists and GP practices [10], but further research is required to investigate the best measures to empower pharmacists in AMS roles.

The objective of this study was to explore the views of pharmacy staff and service users on providing or receiving advice for suspected or confirmed UTIs in the community pharmacy setting. More specifically, this study aimed to identify opportunities to enhance the role of community pharmacists in the management of UTI by exploring the journey of service users with urinary symptoms.

## 2. Methods

### 2.1. Study Design

**Figure 1:**
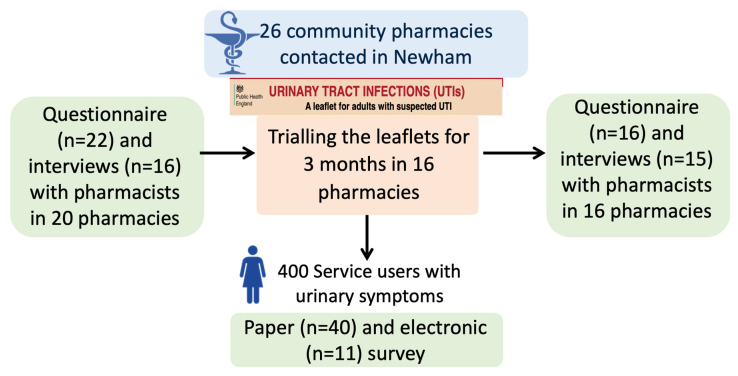
Design of the study.

This study is part of a trial to implement Public Health England’s (PHE) UTI TARGET leaflets [16] in community pharmacies (Figure 1). The TARGET UTI leaflets are designed to be shared with service users to facilitate communication between healthcare professionals and service users and to increase their confidence on self-care [17]. The leaflets follow relevant National Institute for Health and Care Excellence (NICE) guidelines. The study used mixed methods with questionnaires and interviews to explore the views of pharmacists and service users with suspected or confirmed UTI.

### 2.2. Questionnaires and Interviews Schedules

Two semi-structured questionnaires and interview schedules to explore the role of pharmacists in the prevention and management of UTIs (before and after trialling the leaflets) and one semi-structured questionnaire to analyse the opinion of service users were developed by a multidisciplinary team of clinicians and researchers at Imperial College London and PHE (Supplementary Materials S1–S6). The team included doctors specialized in infectious diseases (N.P.S., A.H.), a community pharmacist (P.P.), a clinical microbiologist (C.M.), and researchers specialized in qualitative methodology and implementation science (R.A., L.J., D.L., R.Ah.).

The questionnaires were theoretically informed by the Consolidated Framework for Implementation Research to understand individual level and contextual influencing factors to the adoption of the leaflet [18]. The questionnaires were reviewed and tested by two community pharmacists and were refined according to comments. The pharmacist questionnaires collected information about demographics, characteristics of the pharmacies, particularities of giving advice in the pharmacy setting generally, and specifically about the UTI patient journey, using closed and open-ended questions. The questionnaire for service users collected information about demographics, literacy, the service user experience of their suspected UTI, its management, self-care, and resolution. This was piloted with a service user representative and subsequently revised based on feedback. Interviews were done by a doctor specialized in infectious diseases.

### 2.3. Study Setting: Community Pharmacies in Newham

Community pharmacies were included in one London borough (Newham), purposively sampled, which would enable views from service users of different socio-economic status. An invitation, including an information leaflet and consent form, was sent by the local pharmaceutical committee lead pharmacist to 26 pharmacies in April 2019. Researchers sent non-responders a reminder 2 weeks later.

### 2.4. Participant Enrolment

Pharmacists agreed to participate in a phone interview and complete an electronic questionnaire before trialling the TARGET UTI leaflets for 3 months in their pharmacy. At the end of the 3 months, the pharmacists participated in a second interview and completed a further questionnaire. These pharmacists were provided with a £30 voucher incentive after each interview. Service users who received the TARGET UTI leaflet at the pharmacy were invited to participate in the study in person when they were given the leaflet to complete a paper or electronic survey at home. The paper survey with a written consent form was attached to the leaflet in a prepaid envelope and the electronic survey was accessible via the use of a QR code or a weblink both written on the paper survey. Service users were provided a £10 voucher incentive if they completed the survey electronically or sent it by mail.

### 2.5. Data Analysis

The interviews were recorded, anonymized, and transcribed verbatim by a professional company and checked against the interviews by a researcher. Interviews were analysed by a researcher using an inductive thematic analysis [19]. Two other authors independently and inductively coded three different transcripts. The three researchers collectively reviewed and reached a consensus about the application of themes through independent coding and group discussion, which were then reviewed and agreed by the research team. The interviews were then coded according to the themes using the NVivo 12 software. Data from the closed-ended questions of the surveys were imported into the R software (version 3.2.4). Numerical data were presented as absolute numbers, proportion, median ± interquartile range (IQR). Pearson’s chi-squared tests were used to compare the results among service users.

### 2.6. Ethics

The study received approval from Imperial College London and ethics committee [Imperial College Research Ethics Committee reference: 18IC4777]. Data management was compliant with the European General Data Protection Regulation.

## 3. Results

### 3.1. Participants: Pharmacists and Service Users

Among the 26 pharmacies contacted in Newham, 20 (77%) participated in the pre-intervention assessment. Among them, 16 pharmacies (62%) participated in the study including the pre-intervention questionnaire and interview, the 3-month trial of the leaflets, and the post-intervention questionnaire and interview. Sixteen interviews and 22 questionnaires were completed before and 15 interviews and 16 questionnaires after trialing the leaflet (Figure 1). The median duration of the interviews was 24 min (IQR, 19–27) and 16 min (IQR, 14–17) for the pre- and post-intervention interviews, respectively. The pharmacist participants comprised 8 women and 14 men, with a median of 15 years (IQR, 5–30) post qualification experience (Table 1). Fifty-one service users participated in the survey, the majority of whom were female; 43 (84%). Twenty-one participants (41%) had recurrent UTIs, 23 (45%) had previously experienced one or two UTIs, and 6 (12%) had a UTI for the first time.

### 3.2. Survey Results: The Patient UTI Journey in the Community

**Figure 2:**
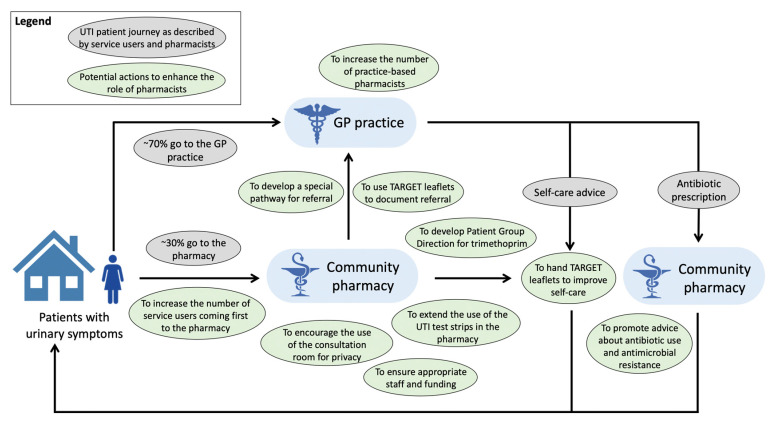
Potential actions to enhance the role of community pharmacists in the management of service users with suspected or confirmed urinary tract infection. GP, general practitioner.

Before seeing a healthcare professional, 15/51 (29%) service users reported consulting their family and 9/51 (18%) reported consulting online sources (e.g., NHS choices) for information on urinary symptoms. Younger participants (aged 18–34 years versus those aged over 34 years) were more likely to access information about UTI on the internet (50% versus 18%) (*p* = 0.01). Two-thirds (n = 31, 62%) of the service users reported drinking more fluids before going to the pharmacy or visiting their GP, but 20 (39%) did not. Some service users reported taking paracetamol (n = 15, 29%), cranberry juice or capsules (n = 12, 24%), resting (n = 10, 20%), cystitis sachets (n = 8, 16%), nonsteroidal anti-inflammatory drugs (n = 7, 14%), or time off work (n = 2, 4%). Cystitis sachets are popular OTC medications in the United Kingdom that contain sodium citrate or potassium citrate and could reduce the acidity of urine. However, there is currently no evidence to support taking cystitis sachets or cranberry products to improve UTI symptoms. Thirty-seven service users (72%) went to a pharmacy following a GP visit after being prescribed an antibiotic and 13 (25%) went to a pharmacy before visiting a GP (Figure 2). The results from the pharmacists’ questionnaires confirmed this finding stating that, on average, 71% of service users came to the pharmacy following a GP visit with an antibiotic prescription and 29% visited the pharmacy first for advice and OTC medication. Service users rarely reported (n = 2, 4%) going to the pharmacy following a GP visit when antibiotics had not been prescribed.

### 3.3. Advice in the Pharmacy

During interviews, pharmacists identified barriers and facilitators to providing healthcare advice in the pharmacy setting compared with a GP practice (Table 2). The most cited barriers were lack of staff or time (n = 17, 77%), language barrier (n = 13, 59%), and absence of access to patient medical records (n = 9, 41%). On the contrary, facilitators were that pharmacists are trained and confident in giving advice (n = 22, 100%), that no appointment is needed (n = 17, 77%), and that pharmacies have long opening hours (n = 14, 64%). The language barrier was mitigated by the number of languages spoken by the pharmacists and their staff: we discussed this point with six pharmacists who all reported the fluent use of four to six Asian languages in their pharmacy in addition to English.

Service users were mostly (n = 33, 65%) comfortable discussing their urinary symptoms with a pharmacist, as long as it was confidential and in private. As expected, the majority of service users (n = 36, 70%) did not want to discuss urinary symptoms at the counter if they could be overheard by other customers.

When asked via questionnaires about the most important self-care advice given to service users with a suspected UTI, all the pharmacists (n = 22) recommended drinking plenty of fluids and taking OTC products (e.g., cystitis sachets, cranberry products), 64% (n = 14) recommended painkillers, 36% (n = 8) discussed red flags to visit the GP, and 27% (n = 6) gave advice regarding preventative care. All pharmacists who were interviewed agreed that it was difficult to give comprehensive self-care advice because of the lack of time. Overall, the pharmacists were confident in discussing UTIs with service users.

### 3.4. Communication with the GP

During pharmacist interviews, the reasons for referral to the GP were explored (Figure 2). The pharmacists referred all male patients, pregnant women, older adults (over 65 years old) or children (below 16 years old), patients with symptoms lasting for more than 48 h, lower abdominal pain or kidney pain, temperature, blood in the urine or severe comorbidities, and those presenting with recurrent UTIs to the GP. However, all pharmacist participants pointed out that they did not have a facilitated way to contact GPs and could only instruct patients to visit their GP:

“So, there’s not much, much of a connection between the two settings. For example, if there was a patient that we were particularly concerned about, we can’t call up the GP practice and say, oh, could you give them early appointment because I’ve seen them and I’ve noticed X, Y, Z. There’s not that rapport yet or there’s not that importance [placed in] a pharmacist’s view, I feel, in community.” P4.

As such, they stressed the need to develop a special referral pathway between pharmacies and GPs (n = 16, 100%) and also suggested that the TARGET UTI leaflets could be used as a referral notice between healthcare professionals.

### 3.5. The Role of Pharmacists

Table 3 presents the themes and sub-themes that were generated from the qualitative analysis of the interviews with the pharmacists regarding the management of service users with suspected or confirmed UTI. Most pharmacists interviewed (n = 13, 81%) suggested that pharmacists could act as a first-line triage for service users with UTIs:

“Well, I think they [the patients] should come first to the pharmacy (…) and then you could screen them and see whether they need to be actually seen by the GP.” P13.

The pharmacists reported that, if they were the first healthcare professional to give advice to service users with suspected or confirmed UTIs, this could relieve pressure on other NHS services, including GPs. However, they requested additional funding and staff to give advice for those with suspected UTI as pharmacies are not currently funded for this. Some also asked for reimbursement for this activity:

“Yeah, but you see having a role to play is one thing but you need to be remunerated for that role. You can’t expect pharmacists to do everything for nothing” P13.

Increasing the patient group directions (PGDs) to prescribe first-line treatments for uncomplicated UTIs could help enhance this role:

“[Having a UTI PGD] …could be something which might take a bit of work off the GPs. And in that service, we do get a consultation fee as well and we do that service for Pharmacy First or the Minor Ailment. So, I think that’s some revenue, it works for the GP and it works for the patient” P3.

All the pharmacists interviewed (n = 16, 100%) agreed that they had a major role in the control of AMR by educating service users about antibiotics and infections for which antibiotics are not needed. They reported giving daily self-care advice for viral infections and self-limiting UTIs, and triaging patients and advising when they should consult a GP. They also described having an important role in preventative measures in community care and participating in preventing bacterial infections by promoting a healthy lifestyle:

“That is where it should all start. We [pharmacy staff] should be highly focused on prevention, and providing advice for people to self-care and self-manage, and improve their health. And that will stop, forget the resistance [AMR], you will not even need to prescribe antibiotics if people are living a healthy lifestyle. (…) So, the biggest impact the pharmacist can make is in the prevention agenda” P10.

## 4. Discussion

### 4.1. Summary

We explored the UTI patient journey with both pharmacists and service users to identify opportunities to enhance the role of community pharmacists specifically in the management of UTIs. The self-care management of service users with suspected UTI can still be improved as 38% of service users did not report drinking plenty of fluids before seeing a healthcare professional. One-fourth of service users with suspected UTI sought help first from a pharmacy, but the majority visited the pharmacy to pick up their antibiotic prescription after a visit to the GP. Barriers to giving advice in the pharmacy were the lack of staff or time, the language barrier, and the absence of access to patient medical records. Pharmacists were trained and confident to give advice to patients with suspected UTIs, but they pointed out the lack of a specific pathway to refer patients who need an antibiotic to GPs. Furthermore, they raised the need for additional funding and staff to enable an increased role of community pharmacists in the management of uncomplicated UTIs. Community pharmacists integrated their role to fight AMR into the wider context of healthcare education and promotion of a healthy lifestyle.

### 4.2. Comparison with Existing Literature

The proportion of service users with urinary symptoms coming first to a pharmacy (25%) is close to what has been found in another study, in which 36% of the patients presented directly to a pharmacy [12]. In a household survey conducted in 2014 in England, only 13% of females who had ever had a UTI reported going first to a pharmacy. The rate in this study may have been lower as it included a wide age range of participants [9]. As described in recent studies, this study found support from both patients and pharmacists for increased access to UTI management and advice through community pharmacies [12,20]. This confirms the need for interventions targeting community pharmacies to improve the UTI patient journey including self-care advice and appropriate referral. According to this study and the literature, the development of a PGD for uncomplicated UTIs is supported by pharmacists in order to extend their management options [12]. A recent study found that a community pharmacy-led UTI test-and-treat service for women aged 16–64 years with urinary symptoms helped to support the appropriate use of antibiotics and reduced demand on other NHS resources such as GP surgeries.^20^ However, there is a need to carefully consider the advantages of PGDs, which should lead to more timely treatment of UTI with the potential drawback of increased use of antibiotics in UTIs, as has been found for chloramphenicol and eye infections [21]. The risk of overuse of antibiotics for suspected UTIs leading to increased AMR could be mitigated with an increased pharmacy access to clear protocols, accurate point-of-care testing [20], urine culture, and shared patients records [22]. Sixty-two percent of the service users in this study took extra fluids before consulting a healthcare professional, as compared with 35% in a study in 2014 in the United Kingdom [9]. This might reflect the effect of recent interventions including online campaigns to promote self-care of UTIs in the community [23,24].

The barriers to giving self-care advice about UTI in the community pharmacy setting confirm findings in a qualitative study with GPs, pharmacists, pharmacy staff, and representatives from pharmacy organisations in England and Wales [22]. Clinicians reported that lack of time or staff and lack of access to medical information were perceived as barriers to giving effective and thorough self-care advice. Overall, this study supports increasing the collaboration between GPs and community pharmacists as advocated in a joint statement by the Royal College of General Practitioners (RCGP) and the Royal Pharmaceutical Society (RPS) [25]. The proposals of this statement include many of the measures that have been asked for by pharmacists in this study such as a greater role for community pharmacy in managing minor conditions, access to health records, and better links between practice-based pharmacists (clinical pharmacists) and community pharmacy. This has also been highlighted in the community pharmacy 2019 to 2024 contractual framework that encourages the development of point-of-care testing in community pharmacies to support efforts to tackle AMR [26].

### 4.3. Strengths and Limitations

The acceptance rate by community pharmacies to participate was high, and we collected information from both pharmacists and service users’ point of views, facilitating triangulation of data. Although patients from white ethnicity were over-represented in the user responses (53% in this sample versus 28% in the borough according to the 2019 Greater London Authority (GLA) housing-led projection), this sample of service users did have participants representing the diversity of Newham borough. Participants’ age was similar to the local population as a whole. Service user questionnaire completion was quite low, as only 13% of 400 questionnaires were returned. However, this return rate is quite usual in any service evaluation by the public and the BAME (Black, Asian, and Minority Ethnic) populations in research are known to be hard to engage [27]. A large part of the participants had recurrent UTIs (41%), which might be explained by an increased willingness of these patients to answer to questionnaires on UTIs. This population of patients could be more informed than the general population of patients with UTI, however, we still found in this study a large proportion of participants, including patients with recurrent UTIs, who did not report applying the standard self-care advice such as drinking plenty of fluids before seeing a healthcare professional. The small sample size, including 22 pharmacists and 51 patients, as well as the risk of recruitment bias when recruiting pharmacists or service users motivated to participate in a study, are recognised. However, qualitative research aims to attain a range of views rather than necessarily obtaining views representative of the general population. In order to strengthen the results of this study, we have planned with PHE to extend the trial of the leaflets in a rural area and to include interviews with service users. The answers obtained in this study may not easily translate to other countries that do not have a government-sponsored universal healthcare system. Indeed, patients in the United Kingdom have free access to the NHS, which may encourage them to visit their GP instead of going to a community pharmacy. Interviewing GPs may also have provided a more comprehensive scope and additional review regarding the role and value of community pharmacists in AMS for UTIs, but this has already been attained in other qualitative studies [17,22].

### 4.4. Implications for Practice

Prescription options for pharmacists, levels of funding, and incentives are areas to explore in policy and contractual developments. Developing a referral pathway is a way to strengthen the link between pharmacists and GPs and to give more importance to the place of pharmacists in the community setting. The referral of patients from pharmacy to GPs could be improved through the use of the TARGET UTI leaflets that highlight the management of patients with suspected UTI [28]. These results also provide evidence for the deployment of clinical pharmacists working in GP surgeries as they can represent a key link to community pharmacists [10] as part of the UK NHS long-term plan for better integration of care [29]. Primary care networks (PCNs) link the local community and community-based health and social care providers, including pharmacies, with constituent GP practices at its core [30]. PCNs intend to make greater use of community pharmacists’ skills and opportunities to engage service users for integrated out-of-hospital care. Expanding the current test and treat programs for UTIs in community pharmacies by strengthening PGDs could be an interesting approach to increase the role of pharmacists. Indeed, in many countries, and particularly low- and middle-income countries, antibiotics are prescribed by community pharmacies without legal and regulatory framework, which could increase inappropriate prescriptions [31]. In some of these countries where access to a doctor is sometimes difficult, supporting and regulating these prescriptions by pharmacists could be a solution to consider. The results of this study, if confirmed, could inform the writing of guidelines for the management of service users with suspected or confirmed UTI in community pharmacies and inspire future strategies and interventions in the community.

## 5. Conclusions

We identified opportunities and potential interventions to improve the management of service users with suspected or confirmed UTI in community pharmacies. The current reconfiguration of primary care in England with primary care networks and integrated care systems could provide a real opportunity for this collaborative working with potential learning for international initiatives.

## Figures and Tables

**Table 1:** Characteristics of the participants. IQR, interquartile range; UTI, urinary tract infection.

Pharmacists	n (%) or Median (IQR) (n = 22)
Women	8 (36)
Job description	
Non-prescribing pharmacists	18 (82)
Prescribing pharmacists	3 (14)
Pharmacy technician*	1 (4)
Years since qualification	15 (5–30)
Years in the pharmacy	9.5 (5–17)
Pharmacy staff (full time equivalent) in the pharmacy	4 (3–5)
Service users per day	55 (40–100)
Estimated percentage of male service users seen in the pharmacy	61 (52–62)
Service users seen in the consultation room per day	8 (5–12)
Estimated number of service users given healthcare advice	20 (16–37)
**Service Users**	**n (%) (n = 51)**
Women	43 (84)
Age	
Children	1 (2) (completed by the mother)
18–24	8 (16)
25–34	10 (20)
35–44	4 (8)
45–54	11 (22)
55–64	7 (14)
65–74	4 (8)
>75	4 (8)
Ethnicity	
White	27 (53)
Asian	14 (27)
Black	4 (8)
Mixed	4 (8)
UTI history	
Recurrent UTIs	21 (41)
One or two prior episodes	23 (45)
No prior episode	6 (12)

* One pharmacy technician was asked by a pharmacist to complete the pre-assessment questionnaire for the pharmacy, but this participant did not participate in the interviews nor the trial of the leaflets.

**Table 2:** Barriers and facilitators of giving advice in the pharmacy raised during interviews.

Pharmacists n = 22 (%)	
Barriers	
Lack of time or staff	17 (77)
Language barrier	13 (59)
No access to the medical record	9 (41)
Not recognised or funded by health authorities	5 (23)
No possibilities to prescribe medication	5 (23)
Outside the scope of expertise of pharmacists	3 (14)
Waiting time is unpredictable for service users	2 (9)
Some service users prefer information from doctors	2 (9)
Facilitators	
Pharmacists are confident and trained in giving advice	22 (100)
No appointment needed	17 (77)
Long opening hours	14 (64)
Ease of access	13 (59)
Multiple languages spoken by the staff	12 (55)
Financial incentive to give additional advice	10 (45)
Availability and use of a consultation room	9 (41)
Close contact with the service users	8 (36)
Flexible time for consultation (no time limit)	6 (27)
Counter assistants and sufficient staff	5 (23)
Local presence/community-based	2 (9)
Possibility to give advice on the phone	2 (9)

**Table 3:** Themes and sub-themes of the qualitative analysis of interviews on the role of pharmacists in the management of service users with suspected or confirmed urinary tract infection. PGD, patient group direction; NHS, National Health Service; AMR, antimicrobial resistance.

Management of Service Users with Suspected or Confirmed Urinary Tract Infection	n (%) (n = 16)
Pharmacists can act as a first-line triage	11 (69)
They need funding or additional staff to do so	10 (62)
They should have more possibilities to prescribe first line treatments (e.g., PGDs)	5 (31)
They can give dipstick tests and check the results	1 (6)
Strengthening the link between pharmacists and GPs	
Development of a special referral pathway between pharmacies and GPs	16 (100)
Self-care advice relieves pressure on the NHS and the GPs	6 (37)
Reducing the spread of AMR	
Education of service users about infections for which antibiotics are not always needed	16 (100)
Screening patients who need to go to the GP	10 (62)
Self-care advice for viral infections and benign bacterial infections	12 (75)
Promotion of a healthy lifestyle	5 (31)

## Supplementary Materials

The following are available online at https://www.mdpi.com/2079-6382/9/9/583/s1. S1. Pre-intervention interview schedule with pharmacists; S2. Post-intervention interview schedule with pharmacists; S3. Post-intervention electronic questionnaire for pharmacists; S4. Pre-intervention electronic questionnaire for pharmacists; S5. Questionnaire for service users who used the leaflets; S6. Survey with service users—full results.
